# Testing dogs in ape-like conditions: the effect of a barrier on dogs’ performance on the object-choice task

**DOI:** 10.1007/s10071-019-01297-8

**Published:** 2019-07-25

**Authors:** Hannah Clark, David A. Leavens

**Affiliations:** School of Psychology, University of Sussex, Falmer, East Sussex BN1 9QH UK

**Keywords:** Object-choice task, Comparative cognition, Dogs, Domestication hypothesis

## Abstract

Recent reviews have found marked procedural and methodological differences in the testing of different taxonomic groups on the object-choice task. One such difference is the imposition of a barrier in the testing environment of nonhuman primates in the form of a cage, necessitated to ensure the experimenter’s safety. Here, we conducted two studies with domestic dogs (*Canis lupus familiaris*) in which we compared the performance of dogs tested from within a child’s playpen and dogs tested without this barrier present. In Study 1, in a within-subjects design, we found no effect of the barrier on dogs’ ability to use a pointing cue, but there was an increase in instances in which dogs failed to choose a cup. In Study 2, in a between-subjects design, dogs tested with a barrier failed to perform above chance, and were also more likely to fail to make a choice. When dogs tested without a barrier made an incorrect response, these were more likely to be incorrect choices than no choice errors. We discuss the implications of these differences in behavioural responses in function of the presence of a barrier and the necessity of ensuring matched conditions when comparing across species.

## Introduction

The differential performances of domestic dogs and nonhuman primates on the Object-Choice Task (OCT), which measures an individual’s ability to follow human gestural cues, have led to phylogenetic theories regarding their respective socio-cognitive abilities. Numerous studies (e.g., Riedel et al. 2008; Virányi et al. 2008) report that domestic dogs possess specialised skills in comprehending human communicative cues, evidenced by their high levels of performance on the Object-Choice Task (OCT). Whether emerging as a by-product of domestication (Hare and Tomasello 2005) or as a result of humans’ active selection for dogs with specific traits (Miklósi et al. 2003), there is a consensus among some researchers that dogs have an evolved ability to follow human gestural cues. In contrast, apes’ poor performance has been attributed to a separation in the primate lineage resulting in this being (among primates) a human-unique ability (e.g., Herrmann et al. 2007; Tomasello and Carpenter 2007; Moll and Tomasello 2007). Several studies directly compared the performance of nonhuman primates and dogs on the OCT, and concluded that apes, for example, are less similar to human infants than dogs in terms of their socio-cognitive abilities due to convergent evolution (Maclean et al. 2017) and lack the ability to understand communicative intentions (Kirchhofer et al. 2012).

Leavens et al. (2019) discussed the prevalence of systematic confounds with species classification in the comparative cognition literature in studies which compare across species. Such confounds are by no means absent from OCT research, and in fact, some have already been addressed. For example, Leavens et al. (2019) discussed the tendency to compare institutionalised apes with non-institutionalised human infants with no regard for the differences that experiential history with humans may confer. Lyn (2010) highlighted this factor of enculturation with specific reference to the OCT, with several studies demonstrating that apes from backgrounds rich in human interaction are able to follow human communicative cues on the OCT (Call and Tomasello 1994; Lyn 2010; Lyn et al. 2010). Russell et al. (2011) age- and sex-matched 20 bonobos and chimpanzees, half of which who had been cross-fostered by humans, and half of which who had been raised in standard nursery conditions, finding significantly better performance by the cross-fostered apes in comprehending human directional cues. Leavens and Clark (2017) listed 43 individual apes that have passed various versions of the OCT task (see their Table 1). Similarly, it has been shown that dogs from backgrounds lacking in interactive exposure to humans perform much more poorly on the OCT (D’Aniello et al. 2017; Lazarowski and Dorman 2015; Udell et al. 2010a), leading Udell et al. (2010b) and Wynne et al. (2008) among others, to emphasise the role of ontogeny in the development of socio-cognitive skills in dogs rather than their being innate (e.g., Kaminski and Nitzchner 2013).

**Table 1:** Study 1 individual subject and performance data

Name	Breed	Sex	Age (years)	First condition	Trials complete barrier condition	Correct trials Barrier condition	Trials complete no barrier condition	Correct trials no barrier condition
Roxy	Labrador Retriever × Poodle	F	4	No barrier	4	0	4	4
Luka	Parson Russell Terrier	F	4	Barrier	4	2	4	3
Trixie	Yorkshire Terrier	F	2	No barrier	4	3	4	1
Mali	Sussex Spaniel	F	2	Barrier	4	4	4	4
Jack	Yorkshire Terrier	M	5	No barrier	4	0	4	4
Charlie	Shih Tzu	M	4	Barrier	4	2	4	2
Stan	Jack Russell Terrier	M	6	No barrier	4	3	4	4
Freddie	Shih Tzu	M		Barrier	4	4	4	2
Topsy	Cocker Spaniel × Poodle	F	2	No barrier	4	0	4	3
Missy	Whippet	F	2	No barrier	4	4	4	2
Toby	Springer Spaniel	M	8	Barrier	4	3	4	3
Lionel	Lhasa Apso	M	4	No barrier	4	1	4	3
Ruby	Lhasa Apso	F	6	Barrier	4	4	4	4
Badger	Border Collie	M	3	Barrier	4	4	4	3
Spock	Jack Russell Terrier	M	2	No barrier	4	3	4	0
Muffins	Lurcher × Spaniel	F	7	Barrier	4	2	4	0
Mabel	Labrador Retriever × Pointer	F	2	No barrier	4	1	4	2
Amber	Mongrel	F	2	No barrier	4	4	4	4
Marley	Labrador Retriever × Pointer	M	13	No barrier	4	4	4	4
Beth	Labrador Retriever × Pointer	F	3	No barrier	4	3	4	3
Solo	Papillon	M	4	No barrier	4	4	4	4
Ruby	Cocker Spaniel × Poodle	F	0	No barrier	4	4	4	4
Bailey	Staffordshire Bull Terrier	M	0	Barrier	4	4	4	4
Bella	Yorkshire Terrier	F	7	Barrier	0		1	0
Choco	Chihuahua × Podenco	F	4	Barrier	4	4	4	4
Bear	Staffordshire Bull Terrier	M	9	No barrier	4	4	4	3
Spike	English Bulldog × Staffordshire Bull Terrier	M	13	Barrier	4	4	4	4
Eric	Staffordshire Bull Terrier	M	10	Barrier	4	4	4	4
Inca	Labrador Retriever	F	3	No barrier	4	4	4	2
Maisie	Jack Russell Terrier	F	5	Barrier	4	2	4	2
Arlo	Springer Spaniel	M	3	Barrier	4	2	4	4
Maisie	Labrador Retriever × Airdale Terrier	F	8	Barrier	4	3	4	4

Leavens et al. (2019) also noted the differences in testing environments when comparing human infants and apes, in that apes are tested from within cages for safety measures, whereas the infants are not. Such a criticism can be extended to research comparing apes with several other species, notably domestic dogs. In fact, Clark et al. (2019a), in a review of 71 published nonhuman primate and dog OCT studies, found that fully 99% of the nonhuman primates tested were tested with a barrier in the testing environment, compared with less than 1% of domestic dogs. These 16 dogs were all subjects in Kirchhofer et al.'s (2012) comparison of nonhuman primate and dog performance, and the portion of their dog sample which were tested with a barrier performed significantly worse than those tested without. Clark et al. (2019b) (under revision) compared the performance of 18-month- and 36-month-old human children tested with and without a barrier on the OCT. These age groups were chosen, because from 14 months of age, human children reliably follow pointing cues (Behne et al. 2012), and therefore, any behavioural differences could reliably be attributed to the manipulation of the presence of a barrier rather than lack of emergence of these skills. As predicted, all of the children performed at ceiling level; however, there were marked differences in the behavioural responses in both age groups, with increased communicative responses compared with acts of direct prehension when a barrier was present. That the children frequently chose to communicate their choice rather than directly lifting the cup, as they chose to do in the “no barrier” condition, suggests that they perceived the barrier as an impediment to their ability to obtain the reward themselves. Thus, as Leavens et al. (2019) argued, it cannot be assumed that such differences in testing environment have no effect on performance.

Here, we present two studies in which we aimed to investigate whether testing pet dogs in “ape-like” conditions would have an effect on their behavioural responses, one in which a within-subjects design is used, and one in which a between-subjects design is used. The difference in the presentations of the conditions between the two studies is a result of Study 2 constituting an opportunistic use of data which was originally collected for a similar study with a different experimental manipulation, for which, owing to experimenter error, the data were unable to be used. Because between-species comparisons typically present dogs with an OCT without a barrier and nonhuman primates through cage barriers (see Clark et al. 2019a), we thought that this was a good opportunity to examine dogs in a between-subjects administration. Miklósi and Soproni (2006) described and defined the features of pointing cues used on the OCT, reporting that representatives of a number of species show differential performance according to the pointing cue used, and suggesting that different points have different levels of salience and demands on memory. Thus, they distinguished between *ipsilateral* and *contralateral* cues-pointing with the hand closest to the target and the hand furthest away, respectively; *dynamic* and *momentary*, where the first is enacted in front of the subject and maintained until the subject makes a choice, and the latter maintained for 1–2 s; and *distal,* where the distance between the finger and the target is greater than 50 cm and proximal, where this distance is less than 40 cm. Therefore, we compared the performance of dogs tested with and without a barrier on an OCT using an *ipsilateral proximal dynamic* pointing cue in Study 1, and a *contralateral proximal dynamic* pointing cue in Study 2. Clark et al. (2019a) found that nonhuman primates tend to be tested with *contralateral* rather than *ipsilateral*, *dynamic* rather than *momentary* and *proximal* rather than *distal,* pointing cues. Dogs, in contrast, tend to be tested with *ipsilateral, distal* cues, with a nearly equal proportion being *momentary* and *dynamic*. The experimental configuration in both studies was such that it would be categorised as a “central” version of the task according to Mulcahy and Hedge’s (2012) distinctions. Mulcahy and Hedge (2012) and Clark et al. (2019a) found that apes are more frequently tested with this version of the OCT, in which the placement of the containers is such that they are close together, and within the direct line of vision of the subject when they look towards a centrally placed experimenter. This is compared to the “peripheral” version, in which the containers are separated by a greater distance and not within the subjects’ direct line of vision. They found that this latter version is more frequently used when testing dogs, and this, they argue, may affect performance, because having the containers within the direct line of vision may distract the subjects’ attention away from the cue owing to the salience of the food reward held within. Hence, in the present study, we also explore the effects of administration of task features more typical of presentations used with nonhuman primates and we predict that, in line with Kirchhofer et al.'s (2012) findings, there will be a negative effect of the barrier on the dogs’ performance on the OCT.

## Study 1: barrier vs. no barrier within-subjects

### Method

#### Subjects

Thirty-two pet dogs (15 male, 17 female) took part in the study. Dogs ranged in age from 4 months to 13 years (*M* = 4.97, *SD* = 3.50) and comprised a variety of breeds (see Table 1 for individual subject data). Dogs were recruited through adverts on social media, word of mouth, and flyers distributed. Although some dogs had taken part in other cognitive tests before, none had previously been tested on an object-choice task. All subjects were tested individually and by an unfamiliar experimenter. Testing took place inside in a community hall, and dogs were randomly assigned to the first condition prior to testing. One dog was excluded from the final analyses, because she failed to complete more than two trials in one condition due to being apparently nervous and unable to settle.

#### Procedure

**Fig. 1:**
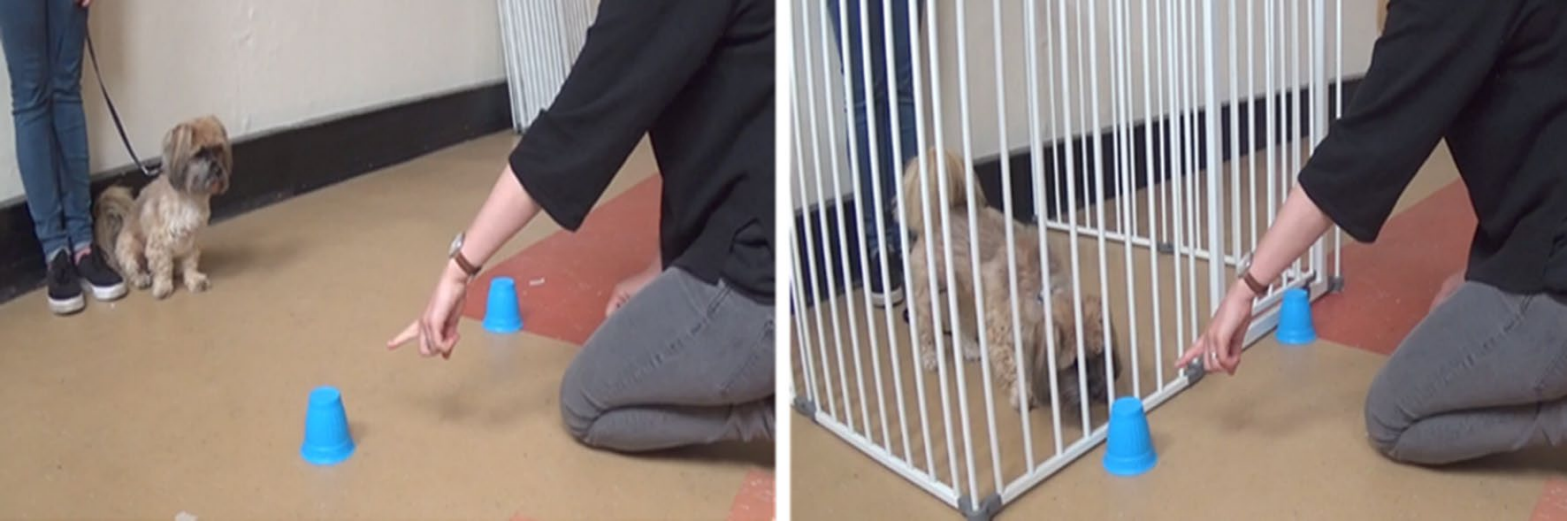
Experimental setup in the two conditions. The owner stood at a distance of 184 cm from the experimenter holding the subject on a 1 m lead, such that the nearest distance between subject and experimenter (depending on the size/position of the subject) was 60 cm. The experimenter was positioned 60 cm from the edge of the barrier in the barrier condition. The distance between the two containers was 60 cm, and the distance between the experimenter’s pointing finger and the container was approximately 10 cm

On arrival at the hall where testing took place, subjects were given time to freely explore the test room, off-lead, to become familiar with the environment. While owners read the information and completed the consent forms, the experimenter interacted with the dog and offered them a treat. When the owner felt that the dog was comfortable and ready to begin, they were asked to put the dog on a 1 m-long lead and to stand in a marked position in the test room (for the barrier condition, this was within a child’s playpen, and for the no barrier condition, this was in the same place but without the playpen present). The experimenter then kneeled in position (see Fig. 1 for experimental setup) and placed the two cups upside down on the floor. She then proceeded to bait both cups with a piece of dry dog food, in sight of the subject, and prior to doing so she called the dog’s name and said “look”, ensuring that the subject was watching, while baiting took place. The experimenter then called the dog’s name, made eye contact and, using an ipsilateral, dynamic point, indicated one of the cups. This cue was held until the subject made a choice. If the subject chose the correct cup, the experimenter gave the subject the piece of food (if they had not already retrieved it themselves), and if the subject chose the incorrect cup, both pieces of food were removed and placed back in the food container. If the subject did not make a choice, the trial continued for 1 min before the experimenter stopped giving the cue and the next trial began. Following recommendations from Udell et al. (2010b), the beginning of the trial was counted from when the cue was presented and the subject released to make a choice. Owners were asked to hold the lead, while baiting took place and to drop the lead, so that the dog was free to move independently as soon as the experimenter pointed. Subjects were given four trials per condition, with the order of conditions counterbalanced prior to testing. The baited container was on the right or left an equal number of times and the order was counterbalanced, such that the container was never on the same side for more than two consecutive trials.

#### Materials and setup

The playpen used in the barrier condition was a Dreambaby Royal Converta 3-in-1 Playpen Gate, measuring 380 × 4 × 74 cm (Dreambaby, Unit 53, Rosyth Business Centre 16 Cromarty Campus, Rosyth, KY11 2WX, Scotland). The containers used to hide the bait were two opaque plastic cups. A premium commercial dry dog food was used for baiting the cups. All dogs were tested on a 1 m-long lead. All testing sessions were recorded on two Sony Handycam HDR-PJ410 video cameras (Sony, 1-7-1 Konan Minato-ku, Tokyo, 108-0075 Japan). The owner stood at a distance of 184 cm from the experimenter holding the subject on a 1 m lead, such that the nearest distance between subject and experimenter (depending on the size/position of the subject) was 60 cm. The experimenter was positioned 60 cm from the edge of the barrier in the barrier condition. The distance between the two containers was 60 cm, and the distance between the experimenter’s pointing finger and the container was approximately 10 cm.

#### Data scoring and analysis

All test sessions were video-recorded and coded at a later date. For each trial, data were recorded for whether a correct choice was made according to Udell et al.’s (2010b) recommendations for the standardisation of OCT tests. Thus, a “correct choice” was defined as the subject first touching or coming within 10 cm of the correct container with their snout. Any other response, including the trial timing out, was marked as an “incorrect response”. Incorrect responses were further categorised into “incorrect choice”, where the subject first touched or went within 10 cm of the incorrect container, and “no choice”, where the subject failed to come into contact with either of the containers before the end of the 1 min trial. Due to a lack of normal distribution in the data, all analyses used nonparametric tests. For correct choices and response types, Wilcoxon’s signed-ranks test was used.

#### Reliability

All trials were coded by the first author, and 20% of the dogs’ trials were coded by a second coder, who was blind to the hypotheses under test (six dogs, with eight trials each, for a total of 48 trials). Inter-observer reliability as to whether each dog was correct on each trial was high: Cohen’s *kappa* = 0.76. In the event of disagreement between coders on a specific trial, the original coding was used.

### Results

#### Correct choices

Dogs performed above chance when tested both without a barrier (binomial test, *p* < 0.001) and when tested with (binomial test, *p* < 0.001). There was no significant difference in percentage of correct choices between the barrier (Mdn = 88%) and the no barrier (Mdn = 100%) conditions, *Z* = − 0.72, *p* = 0.470. This shows that the barrier did not have a suppressing effect on the dogs’ ability to use an ipsilateral proximal dynamic pointing cue on the OCT.

#### Incorrect choice vs. no choice

**Fig. 2:**
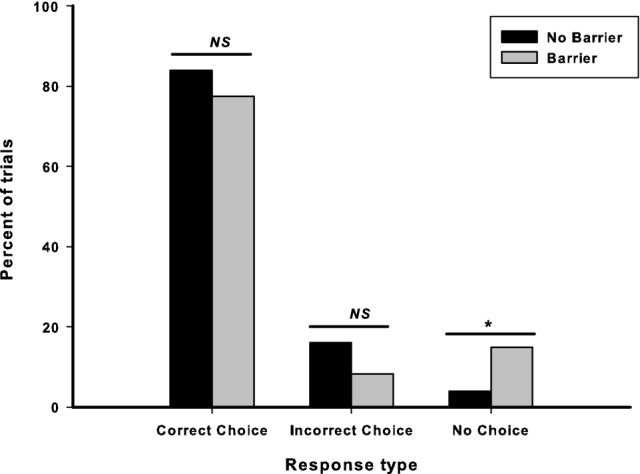
Percentage of trials in which dogs made a correct choice, an incorrect choice and no choice in Study 1. Total number of trials in no barrier condition = 124; total number of trials in barrier condition = 120. *Denotes significant at *p* < 0.05

There was no significant difference in the percentage of incorrect responses that were incorrect choices in between the barrier and no barrier conditions, *Z* = − 1.41, *p* = 0.157. There was a significant difference in the percentage of incorrect responses that were “no choice” responses, with dogs failing to choose one of the cups on significantly more trials in the barrier condition (Mdn = 0%) than in the no barrier condition (no dogs failed to make a choice in this condition), *Z* = − 2.24, *p* = 0.025. This shows that, although there were no significant differences in performance between the two conditions, there were differences in the behavioural responses, with dogs failing to make a choice more when a barrier was present. Figure 2 shows the percentage of correct choices, incorrect choices and no choice responses in the two conditions.

#### Order of administration and side bias

There was no significant effect of order of administration on the proportion of trials correct in the barrier condition (barrier first Mdn = 1.00; no barrier first Mdn = 0.75), Mann–Whitney *U *= 109.00, *p* = .875, or in the no barrier condition (barrier first Mdn = 1.00; no barrier first Mdn = 1.00), Mann–Whitney *U* = 119.00, *p *= 0.965. This shows that the dogs’ performance was not affected by the order of administration of the barrier and no barrier conditions. There was no evidence of a side bias in either the barrier, *Z *= − 1.0, *p *= 0.320, or no barrier, *Z *= − 0.52, *p *= 0.601, conditions.

#### Trial-by-trial analyses

There was no difference in correct choices across trials in the barrier condition, Cochran’s *Q *= 2.33, *p* = 0.506, or in the no barrier condition, Cochran’s *Q *= 2.61, *p* = 0.456. This shows that the dogs’ performance was not affected by successive administrations of the task.

### Discussion

Here, we found that the imposition of a barrier in the testing environment did not affect dogs’ ability to use an ipsilateral dynamic proximal pointing cue on an OCT. There was, however, a subtle difference in the dogs’ behaviour, with significantly more of the incorrect responses comprising “no choice” responses, suggesting that the barrier had a suppressing effect on the frequency of a choice being made.

## Study 2: barrier vs. no barrier between-subjects

### Method

#### Subjects

Thirty-seven (15 male, 19 female) pet dogs took part in the study. Dogs ranged in age from 5 months to 11 years old (*M* = 4.23; *SD* = 2.94) and comprised a variety of breeds (see Table 2 for individual subject data). The dogs were recruited through advertising on social media, word of mouth, and flyers distributed. None of the dogs had previously taken part in an object-choice task study. All testing was completed inside in a community hall by an unfamiliar experimenter. Three dogs were excluded from the final analyses, because they failed to complete at least two trials, due to being inattentive or unable to settle.

**Table 2:** Study 2 subject and performance data

Name	Breed	Sex	Age (years)	Condition	Trials completed	Trials correct
Kiko	Yorkshire Terrier	M	6	Barrier	4	2
Charlie	King Charles Cavalier Spaniel	M	1	Barrier	4	1
Poppy	King Charles Cavalier Spaniel	F	7	Barrier	2	0
Elliot	King Charles Cavalier Spaniel	M	10	Barrier	4	4
Daisy	King Charles Cavalier Spaniel	F	4	Barrier	0	–
Amber	Miniature Dachshund	F	2	Barrier	4	4
Nacho	Chihuahua	M	3	Barrier	4	4
Hoover	Labrador Retriever × Springer Spaniel	M	0	Barrier	4	3
Bo	Setter	M	5	Barrier	4	1
Rupert	Setter	M	6	Barrier	4	4
Chilli	Chilli	F	0	Barrier	4	2
Angel	Siberian Husky	F	4	Barrier	4	2
Missy	Siberian Husky × Staffordshire Bull Terrier	F	4	Barrier	4	1
Kano	Staffordshire Bull Terrier	M	4	Barrier	4	3
Marj	Irish Water Spaniel	F	7	Barrier	4	4
Kobe	Siberian Husky	M	1	Barrier	4	2
Digby	Cocker Spaniel × Poodle	M	2	No barrier	4	1
Phantom	Siberian Husky	M	9	No barrier	4	0
Jet	Siberian Husky × Malamute	M	8	No barrier	4	3
Lucy	Labrador Retriever	F	7	No barrier	4	4
Saffron	Mini Pinscher	F	1	No barrier	4	2
Lucy	Cavalier King Charles Spaniel × Mini Poodle	F	8	No barrier	4	3
Lily	Jack Russell × Shih Tzu	F	5	No barrier	4	2
Lady	Mongrel	F	3	No barrier	4	2
Reggie	Whippet × Collie Greyhound	M	0	No Barrier	4	3
Jax	Rhodesian Ridgeback	M	3	No barrier	4	2
Sandy	Labrador Retriever × Poodle	F	11	No Barrier	4	2
Ronnie	French Bulldog	M	2	No barrier	4	3
Margot	Miniature Dachshund	F	2	No barrier	4	4
Bella	Chihuahua × Jack Russell	F	3	No barrier	4	1
Tommy	Chihuahua	M	5	No barrier	2	0
Baggins	Labrador Retriever	M	5	No barrier	4	4
Alfie	Chihuahua	M	5	No barrier	4	3
Blossom	Chihuahua	F	1	No barrier	3	1

#### Procedure

The procedure was the same as in Study 1, except that the pointing cue used was a contralateral proximal dynamic point. Half of the subjects were tested with a barrier and half without; thus, each dog received four trials in one of the two conditions, barrier or no barrier.

#### Materials

The materials used were the same as in Study 1.

#### Data scoring

The data were scored in the same way as in Study 1.

#### Reliability

All trials were coded by the first author, and 20% of the dogs’ trials were coded by a second coder, who was blind to the hypotheses under test (seven dogs, with four trials each, for a total of 28 trials). Inter-observer reliability as to whether each dog was correct on each trial was high: Cohen’s *kappa* = 0.73. In the event of disagreement between coders on a specific trial, the original coding was maintained.

### Results

#### Correct choices

Dogs tested without a barrier chose the correct container significantly above chance (binomial test, two-tailed, *p* = 0.003). Dogs tested with a barrier did not perform significantly above chance (binomial test, two-tailed, *p* = 0.089). There was no significance difference in the percentage of trials in which the dogs chose the correct container between subjects tested with a barrier (Mdn = 50%) and those tested without a barrier (Mdn = 75%), Mann–Whitney *U* = 88.5, *p* = 0.215. This shows that the barrier had a suppressing effect on the dogs’ ability to use a contralateral proximal dynamic pointing cue.

#### Incorrect choice vs. no choice

**Fig. 3:**
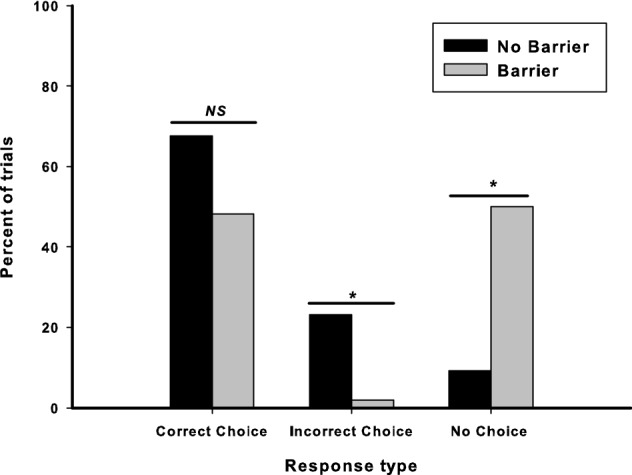
Percentage of trials in which dogs tested with and without a barrier made correct choices, incorrect choices and no choices in Study 2. Total number of trials in no barrier condition = 65; total number of trials in barrier condition = 54. *Denotes significant at *p *< 0.05

Dogs tested without a barrier responded by making an incorrect choice on a significantly greater percentage of trials (Mdn = 25%) than those tested without a barrier (Mdn = 0%), Mann–Whitney *U* = 48.00, *p* = 0.001. Dogs tested with a barrier failed to make a choice on a significantly greater percentage of trials (Mdn = 50%) than those tested without (Mdn = 0%), Mann–Whitney *U* = 60.00, *p* = 0.007. These findings, as in Study 1, show that, although the imposition of a barrier did not lead to a difference in performance, it did elicit different behavioural responses, and increased the likelihood of dogs failing to choose one of the containers. Figure 3 shows the percentage of responses that were correct choices, incorrect choices, and no choice made in the two groups.

#### Side bias

There was no evidence of a side bias, *Z* = − 0.39, *p* = 0.694.

#### Trial-by-trial analyses

There was no significant difference in correct choices made across trials in dogs tested with a barrier, Cochran’s *Q* = 3.33, *p* = 0.343, or dogs tested without a barrier, Cochran’s *Q* = 1.50, *p* = 0.682. This shows that the dogs’ tendency to choose the correct container was not affected by successive administrations.

### Discussion

Here, we found that the presence of a barrier had a suppressing effect on dogs’ ability to use a contralateral proximal dynamic pointing cue on an OCT. This differs from our findings in Study 1, in which dogs performed above chance when tested both with and without a barrier.

As in Study 1, we found a difference in the behavioural responses elicited from the dogs between those tested with and without a barrier. Where incorrect responses were recorded, in the no barrier condition, these tended to be due to subjects choosing the incorrect container, whereas in the barrier condition, they were due to the subjects failing to make a choice. This shows that the presence of a barrier does affect subjects’ responses on the OCT and thus, comparing across groups when one is tested with and the other without a barrier, represents an experimental confound.

## General discussion

In Study 1, we found no effect of the barrier on dogs’ ability to use an ipsilateral proximal dynamic pointing cue on the OCT, whereas in Study 2, we did find that the dogs’ performance was significantly worse on a *contralateral proximal dynamic* pointing cue. The latter finding supports the results of Kirchhofer et al. (2012), who also found a reduction in success rate in dogs tested with a barrier compared to those without. That we did not find this reduction in performance in the first study may be due to the different types of pointing cue used. Miklósi and Soproni (2006); Udell et al. (2013) reported that the ipsilateral proximal dynamic pointing cue is one of the simpler cues to follow, and even dogs with minimal prior exposure to humans can succeed at using this cue on the OCT (Udell et al. 2010a). Clark et al. (2019a) in their review found that dogs’ performance was significantly lower with contralateral than ipsilateral pointing cues and momentary distal points, as used in Kirchhofer et al. (2012) are reportedly more difficult to follow (Miklósi and Soproni 2006; Udell et al. 2013), and therefore, it may be that the increased difficulty of the cue led to an increase in the suppressive effect of the barrier.

In both studies, we found that differential behavioural responses were elicited from the dogs according to whether the barrier was present, specifically in the frequency in which incorrect responses were constituted by incorrect choices or failure to choose a cup. In both studies, there were significantly more instances in which the dogs failed to make a choice. Clark et al. (2019b), (under revision) when testing human children with and without a barrier on the OCT, found an increase in communicative behaviour when the barrier was present, as opposed to acts of direct prehension to obtain the reward. One possible explanation for this comes from Leavens et al.’s (2005) Referential Problem Space; that is, when the barrier was present, the children perceived the containers as out of reach (although they were not) and so chose to communicate with the experimenter to influence her behaviour to receive the reward. In the current study, the dogs, when the barrier was present, may have perceived the containers (and thus the reward) as unobtainable and, therefore, lacking the gestural communicative skills of human children, failed to try to obtain the reward.

In Study 2, a further difference was found concerning the behavioural responses of the dogs with regard to incorrect responses. Here, although there was no significant difference in the number of correct choices, the dogs made incorrect choices significantly more when the barrier was not present than when it was. This could be explained with reference to Mulcahy and Call’s (2009) distraction hypothesis. That is, when the containers are within the subjects’ direct line of vision, and they, therefore, have to look past the containers to attend to the cue, the salience of the container and the reward contained within may distract attention away from the cue being given. Kraus et al. (2014) compared dogs’ performance on a central and a peripheral version of the OCT and found that, when tested with a momentary proximal pointing cue, the dogs’ performance, although still above chance, was significantly lower in the central version, providing support for the distraction hypothesis. In Study 2, we matched the testing conditions in Mulcahy and Call (2009) as closely as possible, by also using the same cue type, and found a 67% success rate when no barrier was present and a 48% success rate when the barrier was present (and thus, the dogs were tested in the same conditions as the apes); the apes in their study had a success rate of 58% on the central version of the task. This suggests that the spatial configuration in the current study also affected the dogs’ responses, supporting the distraction hypothesis of Mulcahy and Call (2009), and further showing that across-species comparisons that do not control for these factors have reduced validity.

One limitation of the current studies is the change in design from a within-subjects design in Study 1 to a between-subjects design in Study 2, owing to opportunistic use of data in the second study. Clark et al. (unpublished data) found that different behavioural responses were elicited from human adults on a pointing production task as a function of whether a between- or within-subjects design was employed; therefore, an alternative explanation for the dogs’ performance differences found in the current studies is that it was the experimental design, rather than the different cue type that led to suppressed performance in Study 2 and thus further research which investigates this is warranted.

The results reported here provide further evidence of the necessity of matching testing conditions when comparing performance across different groups. That we found differences in the behavioural responses of the dogs according to whether or not a barrier was present in the testing environment, together with Clark et al.’s (2019b), (under revision) similar findings with human children, provides support to Leavens et al.’s (2019) arguments that we cannot assume that performance is not affected by differences in the testing environment (also see Hopkins et al. 2013). This is further highlighted by the dogs’ chance-level performance in the barrier condition in Study 2. That behavioural differences were found as a function of the presence of a barrier has important implications for claims of evolved specialised socio-cognitive skills in dogs (e.g., Hare and Tomasello 2005). Such claims are made with reference to an evidence base of OCT studies in which dogs show apparent consistently high levels of performance (e.g., Riedel et al. 2008; Virányi et al. 2008); however, the current studies add to a growing body of research that demonstrates the effects of environmental influences on dogs’ performance (e.g., D’Aniello et al. 2017; Lazarowski and Dorman 2015; Udell et al. 2010a) thus supporting claims for a greater role of ontogeny than is accounted for in domestication theories.

In sum, here, we report an empirical test of predictions from recent reviews of the OCT, confirming that methodological and procedural differences in testing environments in this experimental paradigm may more parsimoniously explain apparent species differences in performances than evolution- or selection-based accounts. Thus, appeals to the effects of domestication on cognitive performance (e.g., Hare and Tomasello 2005) are predicated, in part, on between-species comparisons that have confounded procedural factors, such as the presence of a barrier, with species classification. So-called species differences between apes and dogs in the OCT have not adequately controlled for these systematic confounds; these group differences may be simple artefacts of the radically different protocols administered to dogs, compared with the protocols administered to non-dog species (e.g., Hopkins et al. 2013).
